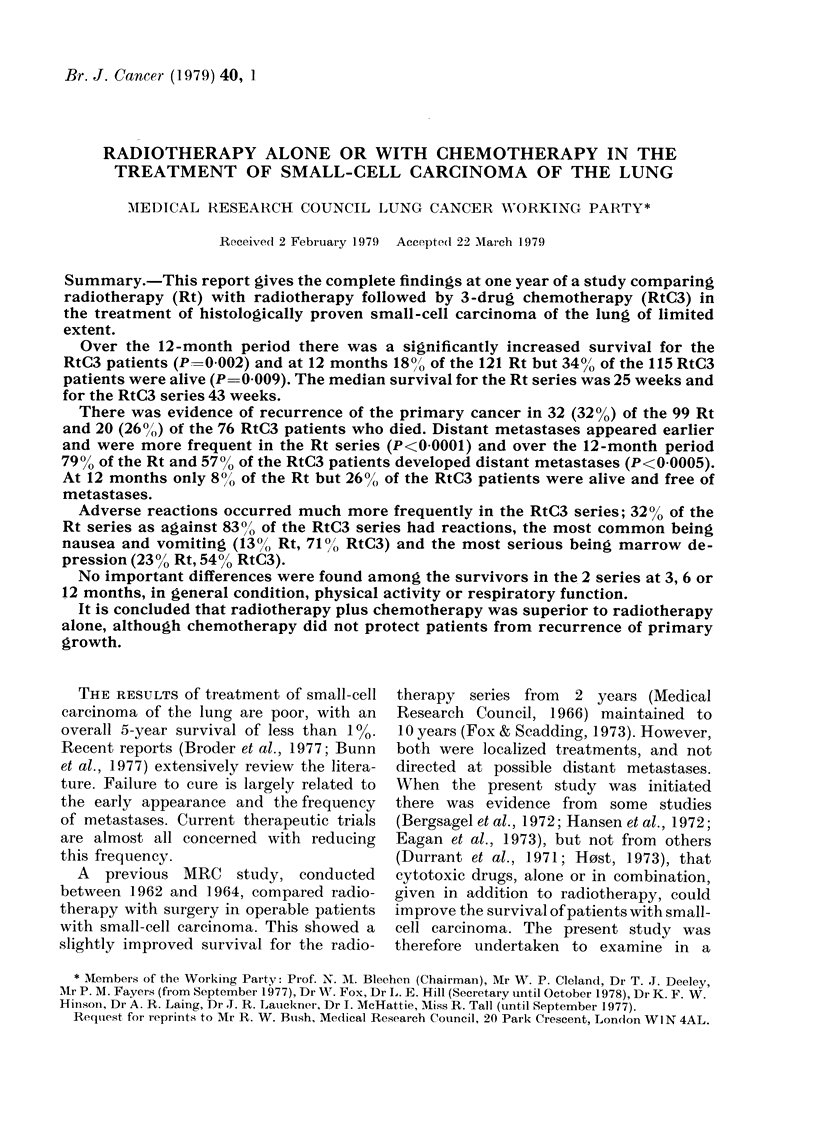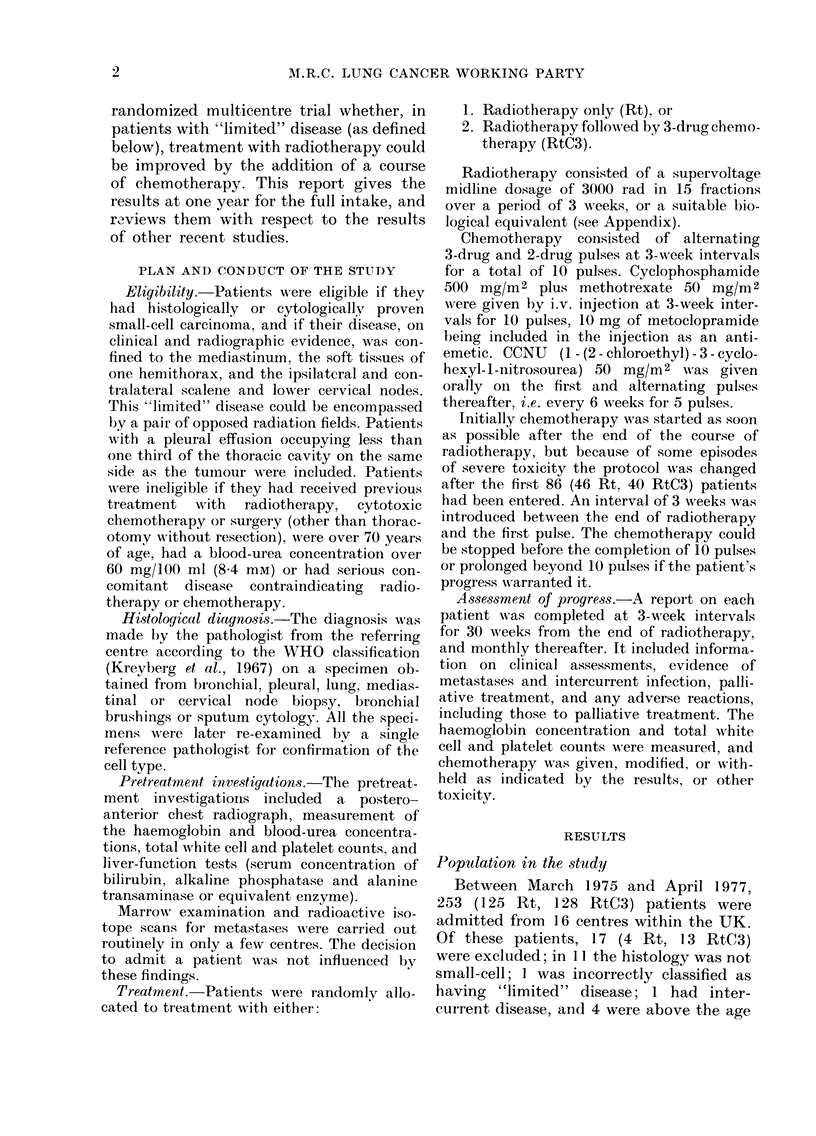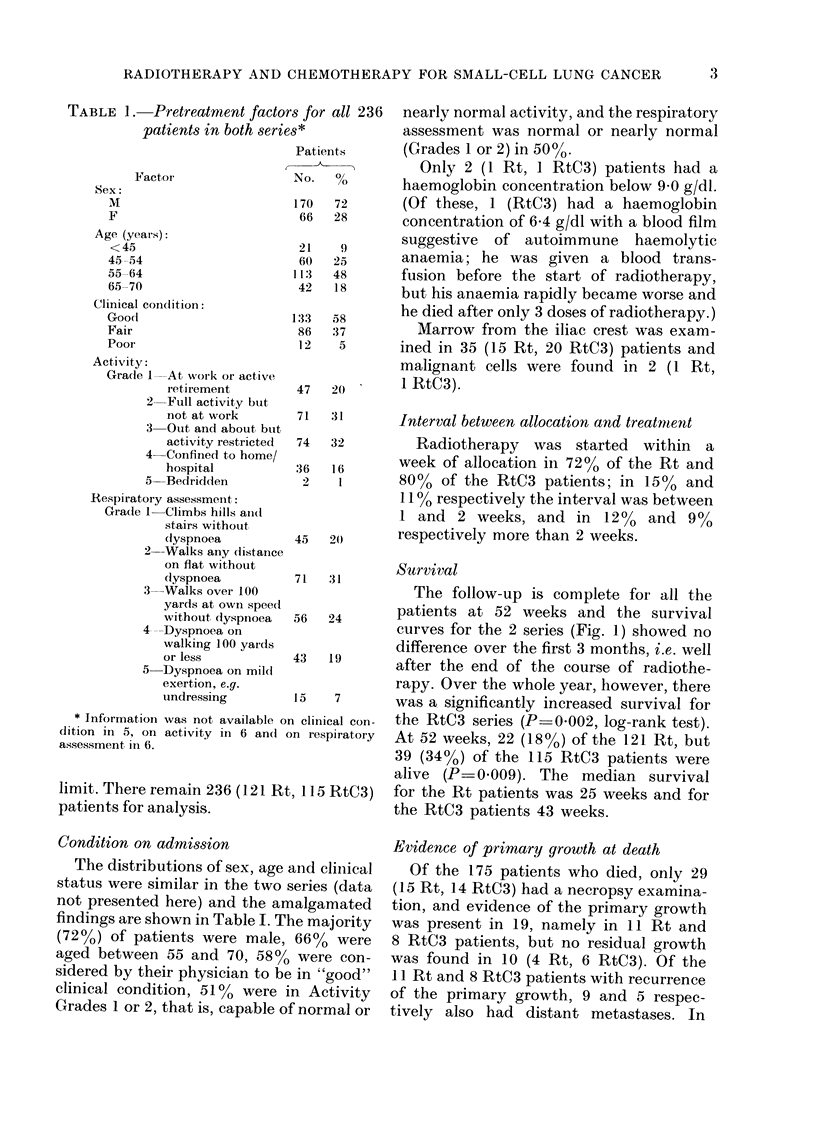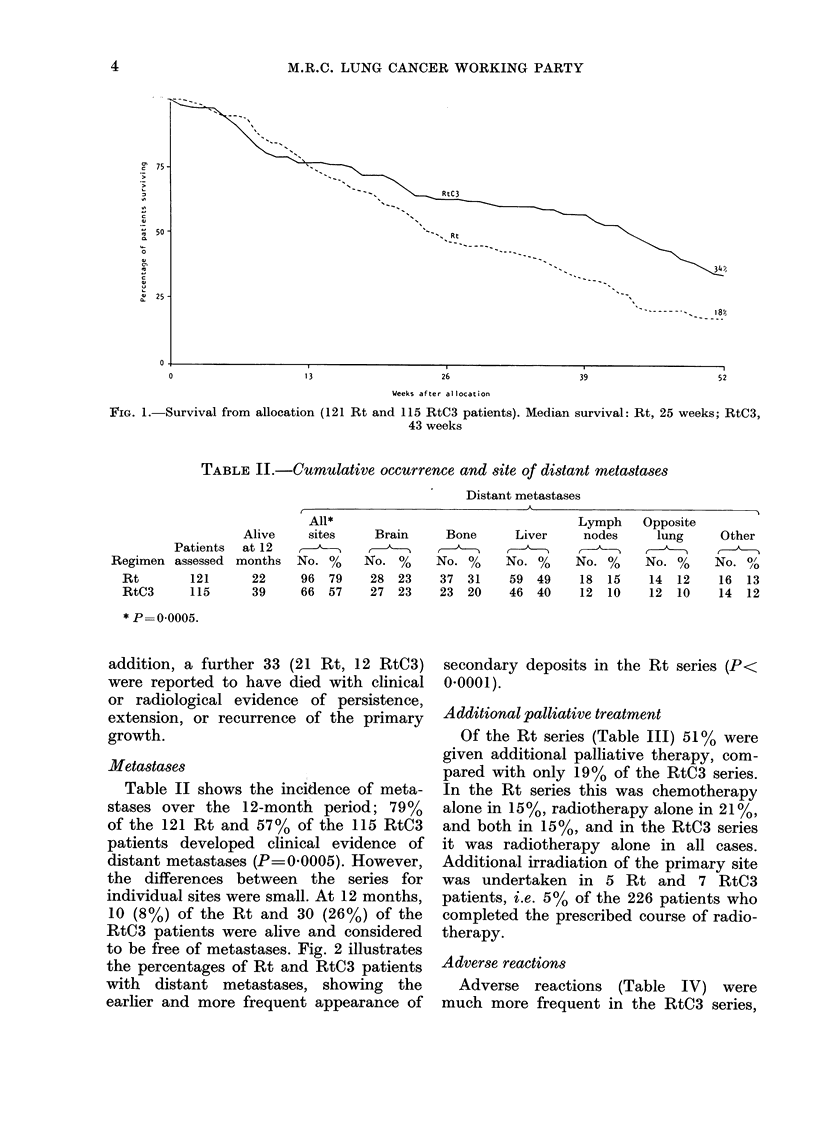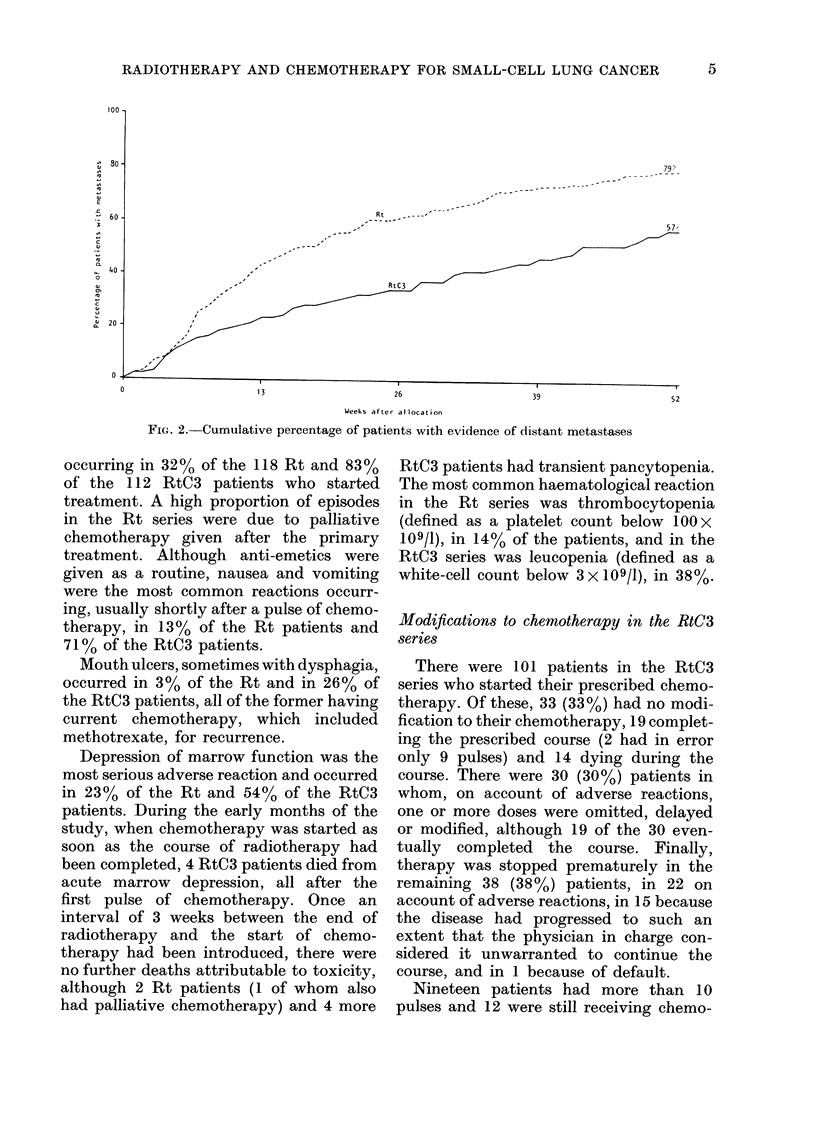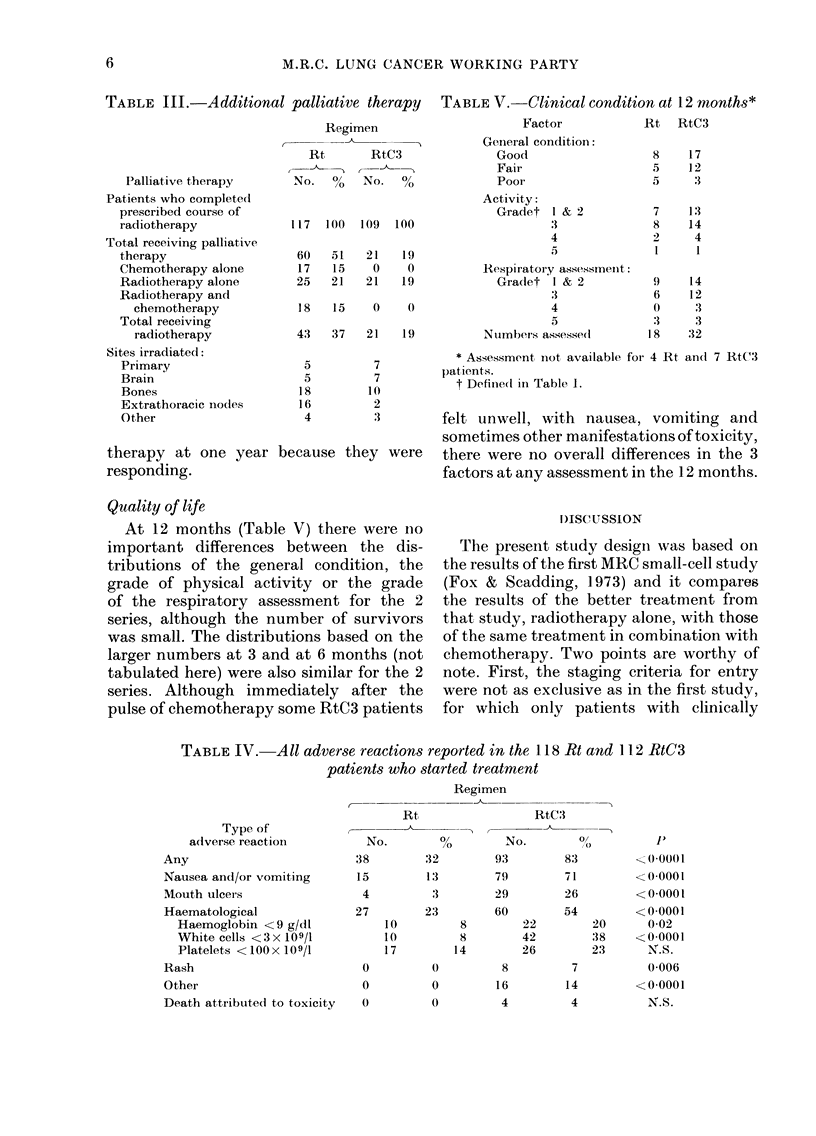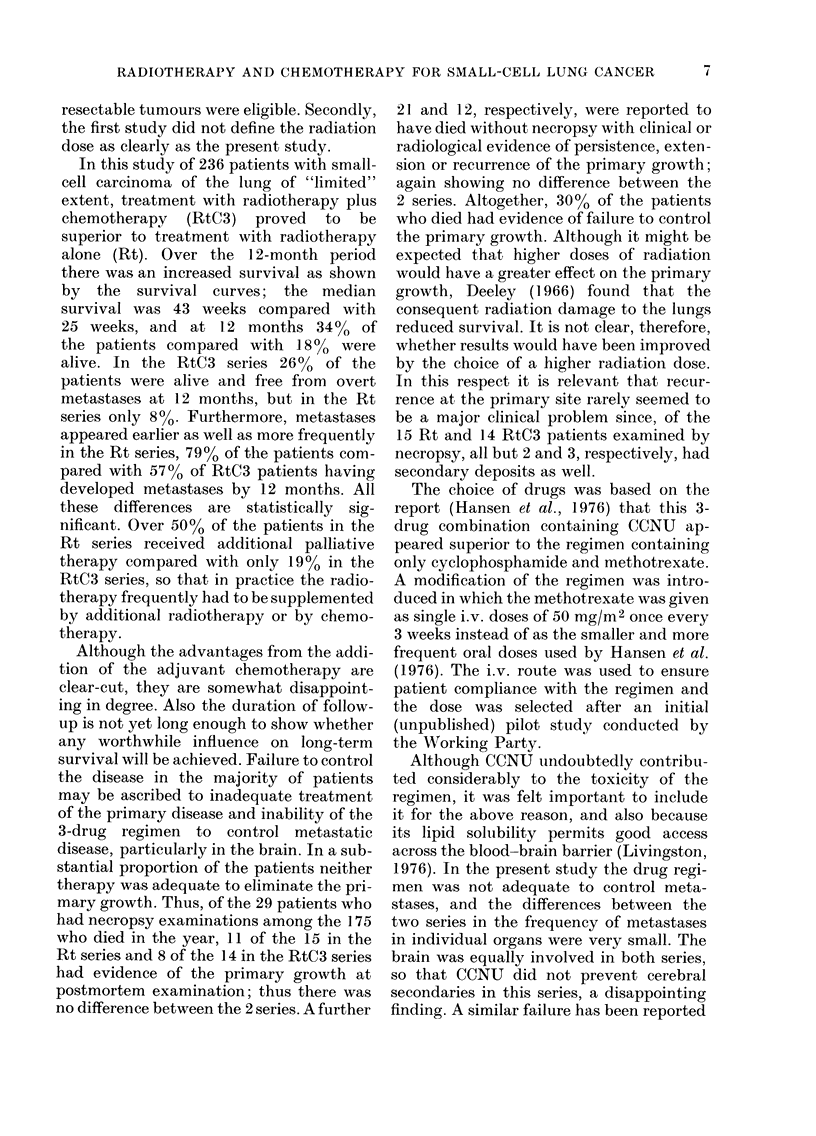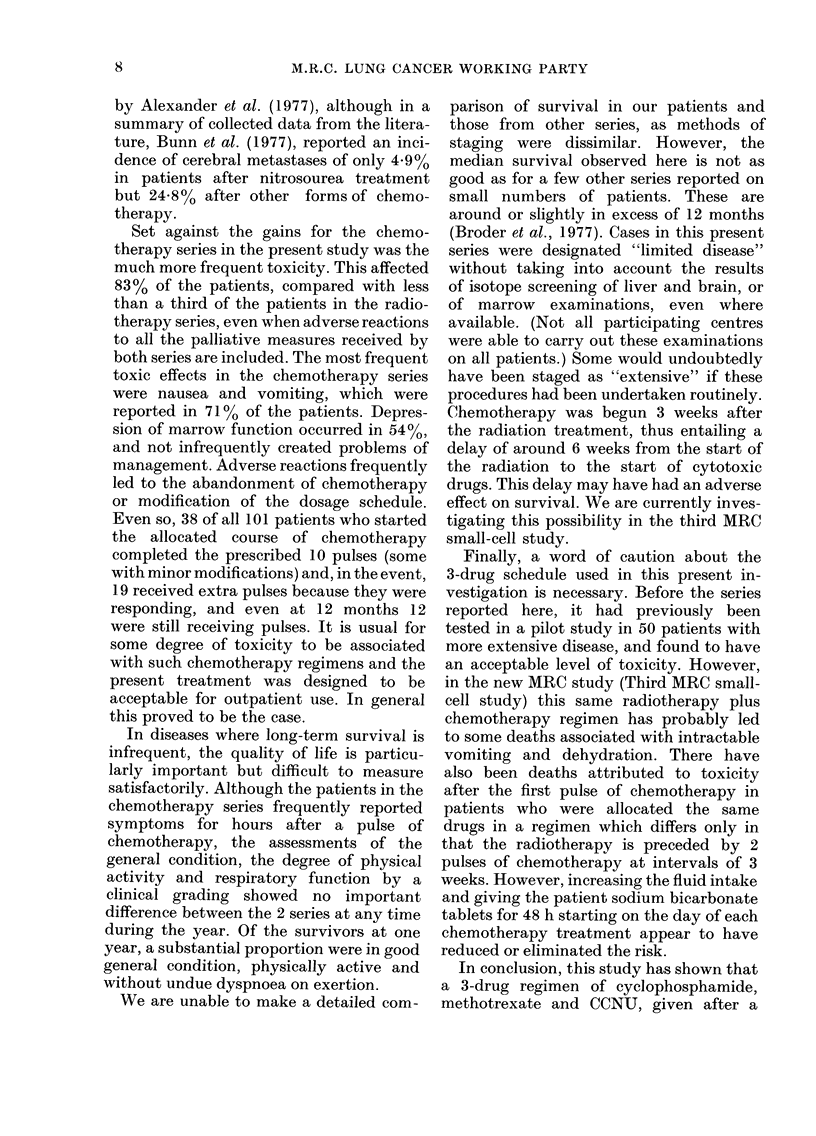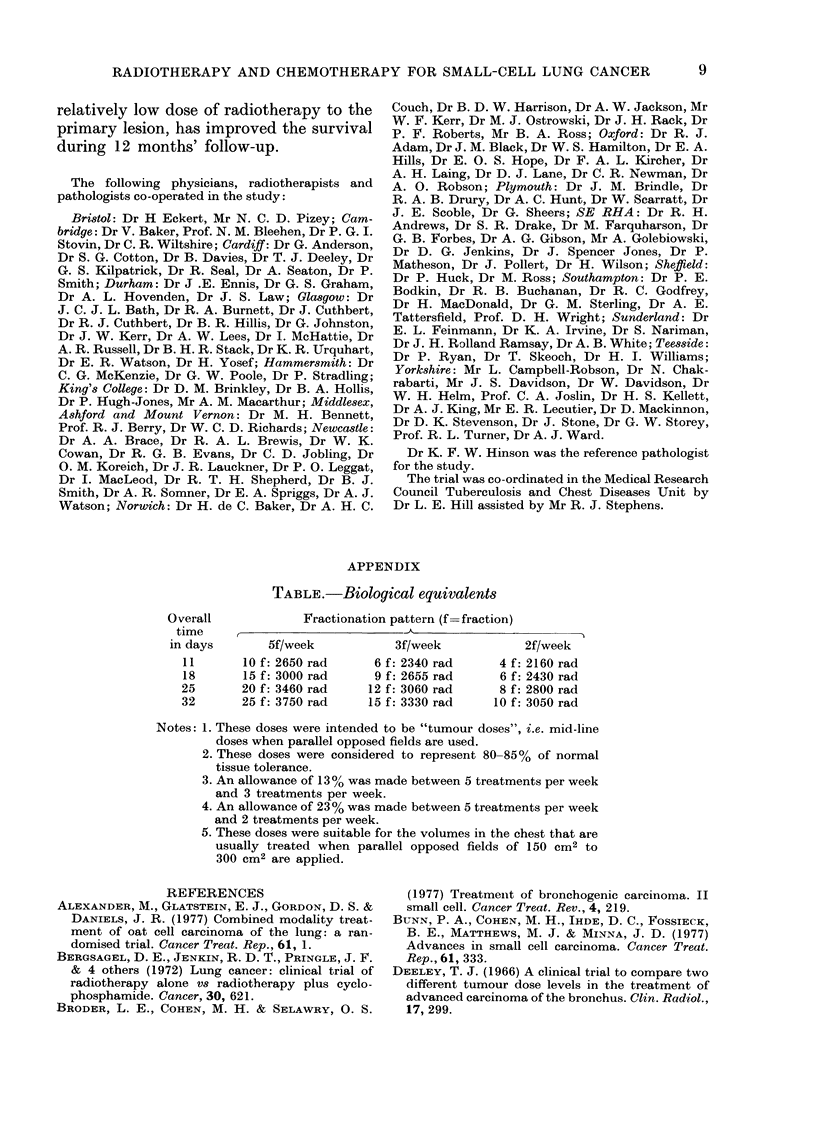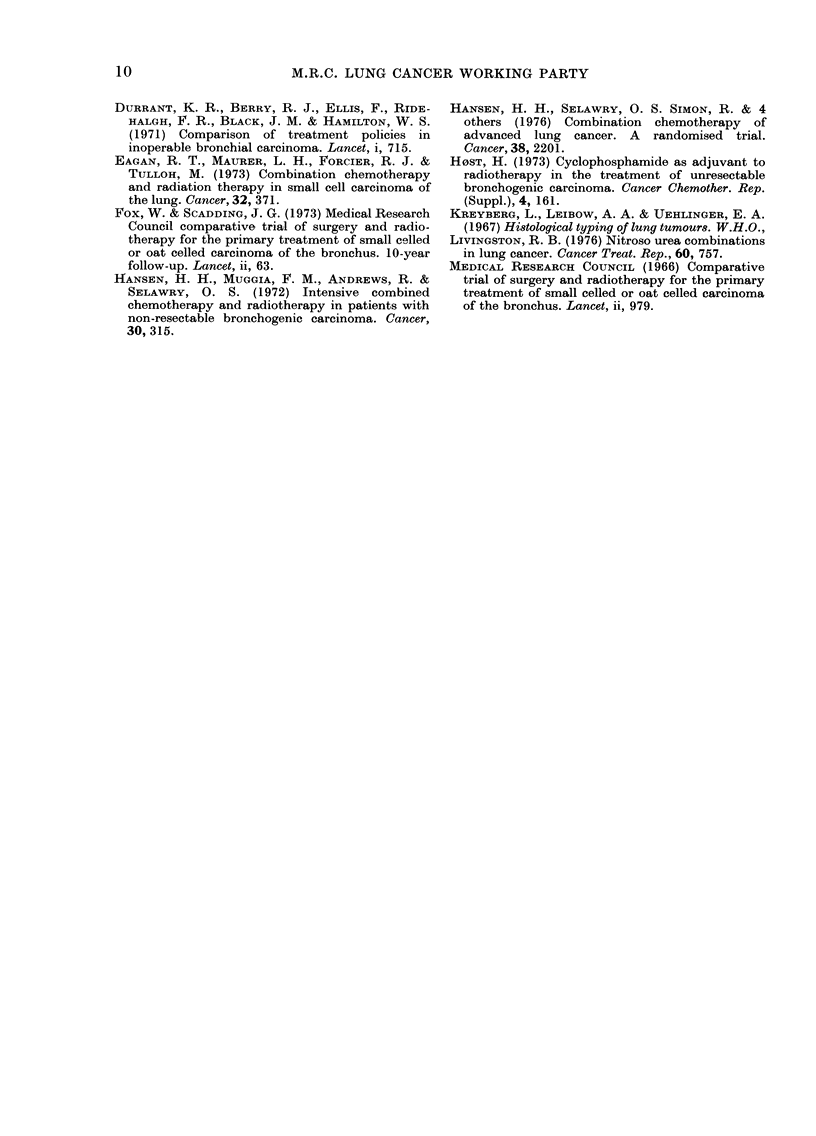# Radiotherapy alone or with chemotherapy in the treatment of small-cell carcinoma of the lung. Medical Research Council Lung Cancer Working Party.

**DOI:** 10.1038/bjc.1979.136

**Published:** 1979-07

**Authors:** 

## Abstract

This report gives the complete findings at one year of a study comparing radiotherapy (Rt) with radiotherapy followed by 3-drug chemotherapy (RtC3) in the treatment of histologically proven small-cell carcinoma of the lung of limited extent. Over the 12-month period there was a significantly increased survival for the RtC3 patients (P = 0.002) and at 12 months 18% of the 121 Rt but 34% of the 115 RtC3 patients were alive (P = 0.009). The median survival for the Rt series was 25 weeks and for the RtC3 series 43 weeks. There was evidence of recurrence of the primary cancer in 32 (32%) of the 99 Rt and 20 (26%) of the 76 RtC3 patients who died. Distant metastases appeared earlier and were more frequent in the Rt series (P less than 0.0001) and over the 12-month period 79% of the Rt and 57% of the RtC3 patients developed distant metastases (P less than 0.0005). At 12 months only 8% of the Rt but 26% of the RtC3 patients were alive and free of metastases. Adverse reactions occurred much more frequently in the RtC3 series; 32% of the Rt series as against 83% of the RtC3 series had reactions, the most common being nausea and vomiting (13% Rt, 71% RtC3) and the most serious being marrow depression (23% Rt, 54% RtC3). No important differences were found among the survivors in the 2 series at 3, 6 or 12 months, in general condition, physical activity or respiratory function. It is concluded that radiotherapy plus chemotherapy was superior to radiotherapy alone, although chemotherapy did not protect patients from recurrence of primary growth.


*Br. J. Cancer* (1979) **40**, 1

### RADIOTHERAPY ALONE OR WITH CHEMOTHERAPY IN THE TREATMENT OF SMALL-CELL CARCINOMA OF THE LUNG

MEDICAL RESEARCH COUNCIL LUNG CANCER WORKING PARTY*

Received 2 February 1979 Accepted 22 March 1979

**Summary.—This report gives the complete findings at one year of a study comparing radiotherapy (Rt) with radiotherapy followed by 3-drug chemotherapy (RtC3) in the treatment of histologically proven small-cell carcinoma of the lung of limited extent.**

**Over the 12-month period there was a significantly increased survival for the RtC3 patients ($P=0{\cdot}002$) and at 12 months 18% of the 121 Rt but 34% of the 115 RtC3 patients were alive ($P=0{\cdot}009$). The median survival for the Rt series was 25 weeks and for the RtC3 series 43 weeks.**

**There was evidence of recurrence of the primary cancer in 32 (32%) of the 99 Rt and 20 (26%) of the 76 RtC3 patients who died. Distant metastases appeared earlier and were more frequent in the Rt series ($P<0{\cdot}0001$) and over the 12-month period 79% of the Rt and 57% of the RtC3 patients developed distant metastases ($P<0{\cdot}0005$). At 12 months only 8% of the Rt but 26% of the RtC3 patients were alive and free of metastases.**

**Adverse reactions occurred much more frequently in the RtC3 series; 32% of the Rt series as against 83% of the RtC3 series had reactions, the most common being nausea and vomiting (13% Rt, 71% RtC3) and the most serious being marrow depression (23% Rt, 54% RtC3).**

**No important differences were found among the survivors in the 2 series at 3, 6 or 12 months, in general condition, physical activity or respiratory function.**

**It is concluded that radiotherapy plus chemotherapy was superior to radiotherapy alone, although chemotherapy did not protect patients from recurrence of primary growth.**

THE RESULTS of treatment of small-cell carcinoma of the lung are poor, with an overall 5-year survival of less than 1%. Recent reports (Broder *et al.*, 1977; Bunn *et al.*, 1977) extensively review the literature. Failure to cure is largely related to the early appearance and the frequency of metastases. Current therapeutic trials are almost all concerned with reducing this frequency.

A previous MRC study, conducted between 1962 and 1964, compared radiotherapy with surgery in operable patients with small-cell carcinoma. This showed a slightly improved survival for the radiotherapy series from 2 years (Medical Research Council, 1966) maintained to 10 years (Fox & Scadding, 1973). However, both were localized treatments, and not directed at possible distant metastases. When the present study was initiated there was evidence from some studies (Bergsagel *et al.*, 1972; Hansen *et al.*, 1972; Eagan *et al.*, 1973), but not from others (Durrant *et al.*, 1971; Høst, 1973), that cytotoxic drugs, alone or in combination, given in addition to radiotherapy, could improve the survival of patients with small-cell carcinoma. The present study was therefore undertaken to examine in a

* Members of the Working Party: Prof. N. M. Bleehen (Chairman), Mr W. P. Cleland, Dr T. J. Deeley, Mr P. M. Fayers (from September 1977), Dr W. Fox, Dr L. E. Hill (Secretary until October 1978), Dr K. F. W. Hinson, Dr A. R. Laing, Dr J. R. Lauckner, Dr I. McHattie, Miss R. Tall (until September 1977).

Request for reprints to Mr R. W. Bush, Medical Research Council, 20 Park Crescent, London W1N 4AL.

randomized multicentre trial whether, in patients with "limited" disease (as defined below), treatment with radiotherapy could be improved by the addition of a course of chemotherapy. This report gives the results at one year for the full intake, and reviews them with respect to the results of other recent studies.

### PLAN AND CONDUCT OF THE STUDY

*Eligibility.*—Patients were eligible if they had histologically or cytologically proven small-cell carcinoma, and if their disease, on clinical and radiographic evidence, was confined to the mediastinum, the soft tissues of one hemithorax, and the ipsilateral and contralateral scalene and lower cervical nodes. This "limited" disease could be encompassed by a pair of opposed radiation fields. Patients with a pleural effusion occupying less than one third of the thoracic cavity on the same side as the tumour were included. Patients were ineligible if they had received previous treatment with radiotherapy, cytotoxic chemotherapy or surgery (other than thoracotomy without resection), were over 70 years of age, had a blood-urea concentration over 60 mg/100 ml (8·4 mM) or had serious concomitant disease contraindicating radiotherapy or chemotherapy.

*Histological diagnosis.*—The diagnosis was made by the pathologist from the referring centre according to the WHO classification (Kreyberg *et al.*, 1967) on a specimen obtained from bronchial, pleural, lung, mediastinal or cervical node biopsy, bronchial brushings or sputum cytology. All the specimens were later re-examined by a single reference pathologist for confirmation of the cell type.

*Pretreatment investigations.*—The pretreatment investigations included a postero-anterior chest radiograph, measurement of the haemoglobin and blood-urea concentrations, total white cell and platelet counts, and liver-function tests (serum concentration of bilirubin, alkaline phosphatase and alanine transaminase or equivalent enzyme).

Marrow examination and radioactive isotope scans for metastases were carried out routinely in only a few centres. The decision to admit a patient was not influenced by these findings.

*Treatment.*—Patients were randomly allocated to treatment with either:

1. Radiotherapy only (Rt), or
2. Radiotherapy followed by 3-drug chemotherapy (RtC3).

Radiotherapy consisted of a supervoltage midline dosage of 3000 rad in 15 fractions over a period of 3 weeks, or a suitable biological equivalent (see Appendix).

Chemotherapy consisted of alternating 3-drug and 2-drug pulses at 3-week intervals for a total of 10 pulses. Cyclophosphamide 500 mg/m$^2$ plus methotrexate 50 mg/m$^2$ were given by i.v. injection at 3-week intervals for 10 pulses, 10 mg of metoclopramide being included in the injection as an anti-emetic. CCNU (1-(2-chloroethyl)-3-cyclohexyl-1-nitrosourea) 50 mg/m$^2$ was given orally on the first and alternating pulses thereafter, *i.e.* every 6 weeks for 5 pulses.

Initially chemotherapy was started as soon as possible after the end of the course of radiotherapy, but because of some episodes of severe toxicity the protocol was changed after the first 86 (46 Rt, 40 RtC3) patients had been entered. An interval of 3 weeks was introduced between the end of radiotherapy and the first pulse. The chemotherapy could be stopped before the completion of 10 pulses or prolonged beyond 10 pulses if the patient's progress warranted it.

*Assessment of progress.*—A report on each patient was completed at 3-week intervals for 30 weeks from the end of radiotherapy, and monthly thereafter. It included information on clinical assessments, evidence of metastases and intercurrent infection, palliative treatment, and any adverse reactions, including those to palliative treatment. The haemoglobin concentration and total white cell and platelet counts were measured, and chemotherapy was given, modified, or withheld as indicated by the results, or other toxicity.

### RESULTS

### *Population in the study*

Between March 1975 and April 1977, 253 (125 Rt, 128 RtC3) patients were admitted from 16 centres within the UK. Of these patients, 17 (4 Rt, 13 RtC3) were excluded; in 11 the histology was not small-cell; 1 was incorrectly classified as having "limited" disease; 1 had intercurrent disease, and 4 were above the age

TABLE 1.—*Pretreatment factors for all* 236 *patients in both series**

| Factor | Patients No. | % |
|---|---|---|
| Sex: | | |
| M | 170 | 72 |
| F | 66 | 28 |
| Age (years): | | |
| <45 | 21 | 9 |
| 45–54 | 60 | 25 |
| 55–64 | 113 | 48 |
| 65–70 | 42 | 18 |
| Clinical condition: | | |
| Good | 133 | 58 |
| Fair | 86 | 37 |
| Poor | 12 | 5 |
| Activity: | | |
| Grade 1—At work or active retirement | 47 | 20 |
| 2—Full activity but not at work | 71 | 31 |
| 3—Out and about but activity restricted | 74 | 32 |
| 4—Confined to home/hospital | 36 | 16 |
| 5—Bedridden | 2 | 1 |
| Respiratory assessment: | | |
| Grade 1—Climbs hills and stairs without dyspnoea | 45 | 20 |
| 2—Walks any distance on flat without dyspnoea | 71 | 31 |
| 3—Walks over 100 yards at own speed without dyspnoea | 56 | 24 |
| 4—Dyspnoea on walking 100 yards or less | 43 | 19 |
| 5—Dyspnoea on mild exertion, *e.g.* undressing | 15 | 7 |

* Information was not available on clinical condition in 5, on activity in 6 and on respiratory assessment in 6.

limit. There remain 236 (121 Rt, 115 RtC3) patients for analysis.

*Condition on admission*

The distributions of sex, age and clinical status were similar in the two series (data not presented here) and the amalgamated findings are shown in Table I. The majority (72%) of patients were male, 66% were aged between 55 and 70, 58% were considered by their physician to be in "good" clinical condition, 51% were in Activity Grades 1 or 2, that is, capable of normal or nearly normal activity, and the respiratory assessment was normal or nearly normal (Grades 1 or 2) in 50%.

Only 2 (1 Rt, 1 RtC3) patients had a haemoglobin concentration below 9·0 g/dl. (Of these, 1 (RtC3) had a haemoglobin concentration of 6·4 g/dl with a blood film suggestive of autoimmune haemolytic anaemia; he was given a blood transfusion before the start of radiotherapy, but his anaemia rapidly became worse and he died after only 3 doses of radiotherapy.)

Marrow from the iliac crest was examined in 35 (15 Rt, 20 RtC3) patients and malignant cells were found in 2 (1 Rt, 1 RtC3).

*Interval between allocation and treatment*

Radiotherapy was started within a week of allocation in 72% of the Rt and 80% of the RtC3 patients; in 15% and 11% respectively the interval was between 1 and 2 weeks, and in 12% and 9% respectively more than 2 weeks.

*Survival*

The follow-up is complete for all the patients at 52 weeks and the survival curves for the 2 series (Fig. 1) showed no difference over the first 3 months, *i.e.* well after the end of the course of radiotherapy. Over the whole year, however, there was a significantly increased survival for the RtC3 series ($P=0{\cdot}002$, log-rank test). At 52 weeks, 22 (18%) of the 121 Rt, but 39 (34%) of the 115 RtC3 patients were alive ($P=0{\cdot}009$). The median survival for the Rt patients was 25 weeks and for the RtC3 patients 43 weeks.

*Evidence of primary growth at death*

Of the 175 patients who died, only 29 (15 Rt, 14 RtC3) had a necropsy examination, and evidence of the primary growth was present in 19, namely in 11 Rt and 8 RtC3 patients, but no residual growth was found in 10 (4 Rt, 6 RtC3). Of the 11 Rt and 8 RtC3 patients with recurrence of the primary growth, 9 and 5 respectively also had distant metastases. In

FIG. 1.—Survival from allocation (121 Rt and 115 RtC3 patients). Median survival: Rt, 25 weeks; RtC3, 43 weeks

TABLE II.—*Cumulative occurrence and site of distant metastases*

| | | | Distant metastases | | | | | | | | | | | | | |
|---|---|---|---|---|---|---|---|---|---|---|---|---|---|---|---|---|
| | | | All* sites | | Brain | | Bone | | Liver | | Lymph nodes | | Opposite lung | | Other | |
| Regimen | Patients assessed | Alive at 12 months | No. | % | No. | % | No. | % | No. | % | No. | % | No. | % | No. | % |
| Rt | 121 | 22 | 96 | 79 | 28 | 23 | 37 | 31 | 59 | 49 | 18 | 15 | 14 | 12 | 16 | 13 |
| RtC3 | 115 | 39 | 66 | 57 | 27 | 23 | 23 | 20 | 46 | 40 | 12 | 10 | 12 | 10 | 14 | 12 |

* $P=0{\cdot}0005$.

addition, a further 33 (21 Rt, 12 RtC3) were reported to have died with clinical or radiological evidence of persistence, extension, or recurrence of the primary growth.

*Metastases*

Table II shows the incidence of metastases over the 12-month period; 79% of the 121 Rt and 57% of the 115 RtC3 patients developed clinical evidence of distant metastases ($P=0{\cdot}0005$). However, the differences between the series for individual sites were small. At 12 months, 10 (8%) of the Rt and 30 (26%) of the RtC3 patients were alive and considered to be free of metastases. Fig. 2 illustrates the percentages of Rt and RtC3 patients with distant metastases, showing the earlier and more frequent appearance of secondary deposits in the Rt series ($P<0{\cdot}0001$).

*Additional palliative treatment*

Of the Rt series (Table III) 51% were given additional palliative therapy, compared with only 19% of the RtC3 series. In the Rt series this was chemotherapy alone in 15%, radiotherapy alone in 21%, and both in 15%, and in the RtC3 series it was radiotherapy alone in all cases. Additional irradiation of the primary site was undertaken in 5 Rt and 7 RtC3 patients, *i.e.* 5% of the 226 patients who completed the prescribed course of radiotherapy.

*Adverse reactions*

Adverse reactions (Table IV) were much more frequent in the RtC3 series,

FIG. 2.—Cumulative percentage of patients with evidence of distant metastases occurring in 32% of the 118 Rt and 83% of the 112 RtC3 patients who started treatment. A high proportion of episodes in the Rt series were due to palliative chemotherapy given after the primary treatment. Although anti-emetics were given as a routine, nausea and vomiting were the most common reactions occurring, usually shortly after a pulse of chemotherapy, in 13% of the Rt patients and 71% of the RtC3 patients.

Mouth ulcers, sometimes with dysphagia, occurred in 3% of the Rt and in 26% of the RtC3 patients, all of the former having current chemotherapy, which included methotrexate, for recurrence.

Depression of marrow function was the most serious adverse reaction and occurred in 23% of the Rt and 54% of the RtC3 patients. During the early months of the study, when chemotherapy was started as soon as the course of radiotherapy had been completed, 4 RtC3 patients died from acute marrow depression, all after the first pulse of chemotherapy. Once an interval of 3 weeks between the end of radiotherapy and the start of chemotherapy had been introduced, there were no further deaths attributable to toxicity, although 2 Rt patients (1 of whom also had palliative chemotherapy) and 4 more RtC3 patients had transient pancytopenia. The most common haematological reaction in the Rt series was thrombocytopenia (defined as a platelet count below $100 \times 10^9$/l), in 14% of the patients, and in the RtC3 series was leucopenia (defined as a white-cell count below $3 \times 10^9$/l), in 38%.

*Modifications to chemotherapy in the RtC3 series*

There were 101 patients in the RtC3 series who started their prescribed chemotherapy. Of these, 33 (33%) had no modification to their chemotherapy, 19 completing the prescribed course (2 had in error only 9 pulses) and 14 dying during the course. There were 30 (30%) patients in whom, on account of adverse reactions, one or more doses were omitted, delayed or modified, although 19 of the 30 eventually completed the course. Finally, therapy was stopped prematurely in the remaining 38 (38%) patients, in 22 on account of adverse reactions, in 15 because the disease had progressed to such an extent that the physician in charge considered it unwarranted to continue the course, and in 1 because of default.

Nineteen patients had more than 10 pulses and 12 were still receiving chemo-

TABLE III.—*Additional palliative therapy*

| | Regimen | | | |
|---|---|---|---|---|
| | Rt | | RtC3 | |
| Palliative therapy | No. | % | No. | % |
| Patients who completed prescribed course of radiotherapy | 117 | 100 | 109 | 100 |
| Total receiving palliative therapy | 60 | 51 | 21 | 19 |
| Chemotherapy alone | 17 | 15 | 0 | 0 |
| Radiotherapy alone | 25 | 21 | 21 | 19 |
| Radiotherapy and chemotherapy | 18 | 15 | 0 | 0 |
| Total receiving radiotherapy | 43 | 37 | 21 | 19 |
| Sites irradiated: | | | | |
| Primary | 5 | | 7 | |
| Brain | 5 | | 7 | |
| Bones | 18 | | 10 | |
| Extrathoracic nodes | 16 | | 2 | |
| Other | 4 | | 3 | |

therapy at one year because they were responding.

*Quality of life*

At 12 months (Table V) there were no important differences between the distributions of the general condition, the grade of physical activity or the grade of the respiratory assessment for the 2 series, although the number of survivors was small. The distributions based on the larger numbers at 3 and at 6 months (not tabulated here) were also similar for the 2 series. Although immediately after the pulse of chemotherapy some RtC3 patients felt unwell, with nausea, vomiting and sometimes other manifestations of toxicity, there were no overall differences in the 3 factors at any assessment in the 12 months.

TABLE V.—*Clinical condition at* 12 *months**

| Factor | Rt | RtC3 |
|---|---|---|
| General condition: | | |
| Good | 8 | 17 |
| Fair | 5 | 12 |
| Poor | 5 | 3 |
| Activity: | | |
| Grade† 1 & 2 | 7 | 13 |
| 3 | 8 | 14 |
| 4 | 2 | 4 |
| 5 | 1 | 1 |
| Respiratory assessment: | | |
| Grade† 1 & 2 | 9 | 14 |
| 3 | 6 | 12 |
| 4 | 0 | 3 |
| 5 | 3 | 3 |
| Numbers assessed | 18 | 32 |

* Assessment not available for 4 Rt and 7 RtC3 patients.

† Defined in Table I.

DISCUSSION

The present study design was based on the results of the first MRC small-cell study (Fox & Scadding, 1973) and it compares the results of the better treatment from that study, radiotherapy alone, with those of the same treatment in combination with chemotherapy. Two points are worthy of note. First, the staging criteria for entry were not as exclusive as in the first study, for which only patients with clinically

TABLE IV.—*All adverse reactions reported in the* 118 *Rt and* 112 *RtC*3 *patients who started treatment*

| Type of adverse reaction | Rt No. | | Rt % | | RtC3 No. | | RtC3 % | | P |
|---|---|---|---|---|---|---|---|---|---|
| Any | 38 | | 32 | | 93 | | 83 | | <0·0001 |
| Nausea and/or vomiting | 15 | | 13 | | 79 | | 71 | | <0·0001 |
| Mouth ulcers | 4 | | 3 | | 29 | | 26 | | <0·0001 |
| Haematological | 27 | | 23 | | 60 | | 54 | | <0·0001 |
| Haemoglobin <9 g/dl | | 10 | | 8 | | 22 | | 20 | 0·02 |
| White cells $<3\times10^9$/l | | 10 | | 8 | | 42 | | 38 | <0·0001 |
| Platelets $<100\times10^9$/l | | 17 | | 14 | | 26 | | 23 | N.S. |
| Rash | 0 | | 0 | | 8 | | 7 | | 0·006 |
| Other | 0 | | 0 | | 16 | | 14 | | <0·0001 |
| Death attributed to toxicity | 0 | | 0 | | 4 | | 4 | | N.S. |

resectable tumours were eligible. Secondly, the first study did not define the radiation dose as clearly as the present study.

In this study of 236 patients with small-cell carcinoma of the lung of "limited" extent, treatment with radiotherapy plus chemotherapy (RtC3) proved to be superior to treatment with radiotherapy alone (Rt). Over the 12-month period there was an increased survival as shown by the survival curves; the median survival was 43 weeks compared with 25 weeks, and at 12 months 34% of the patients compared with 18% were alive. In the RtC3 series 26% of the patients were alive and free from overt metastases at 12 months, but in the Rt series only 8%. Furthermore, metastases appeared earlier as well as more frequently in the Rt series, 79% of the patients compared with 57% of RtC3 patients having developed metastases by 12 months. All these differences are statistically significant. Over 50% of the patients in the Rt series received additional palliative therapy compared with only 19% in the RtC3 series, so that in practice the radiotherapy frequently had to be supplemented by additional radiotherapy or by chemotherapy.

Although the advantages from the addition of the adjuvant chemotherapy are clear-cut, they are somewhat disappointing in degree. Also the duration of follow-up is not yet long enough to show whether any worthwhile influence on long-term survival will be achieved. Failure to control the disease in the majority of patients may be ascribed to inadequate treatment of the primary disease and inability of the 3-drug regimen to control metastatic disease, particularly in the brain. In a substantial proportion of the patients neither therapy was adequate to eliminate the primary growth. Thus, of the 29 patients who had necropsy examinations among the 175 who died in the year, 11 of the 15 in the Rt series and 8 of the 14 in the RtC3 series had evidence of the primary growth at postmortem examination; thus there was no difference between the 2 series. A further 21 and 12, respectively, were reported to have died without necropsy with clinical or radiological evidence of persistence, extension or recurrence of the primary growth; again showing no difference between the 2 series. Altogether, 30% of the patients who died had evidence of failure to control the primary growth. Although it might be expected that higher doses of radiation would have a greater effect on the primary growth, Deeley (1966) found that the consequent radiation damage to the lungs reduced survival. It is not clear, therefore, whether results would have been improved by the choice of a higher radiation dose. In this respect it is relevant that recurrence at the primary site rarely seemed to be a major clinical problem since, of the 15 Rt and 14 RtC3 patients examined by necropsy, all but 2 and 3, respectively, had secondary deposits as well.

The choice of drugs was based on the report (Hansen *et al.*, 1976) that this 3-drug combination containing CCNU appeared superior to the regimen containing only cyclophosphamide and methotrexate. A modification of the regimen was introduced in which the methotrexate was given as single i.v. doses of 50 mg/m$^2$ once every 3 weeks instead of as the smaller and more frequent oral doses used by Hansen *et al.* (1976). The i.v. route was used to ensure patient compliance with the regimen and the dose was selected after an initial (unpublished) pilot study conducted by the Working Party.

Although CCNU undoubtedly contributed considerably to the toxicity of the regimen, it was felt important to include it for the above reason, and also because its lipid solubility permits good access across the blood–brain barrier (Livingston, 1976). In the present study the drug regimen was not adequate to control metastases, and the differences between the two series in the frequency of metastases in individual organs were very small. The brain was equally involved in both series, so that CCNU did not prevent cerebral secondaries in this series, a disappointing finding. A similar failure has been reported by Alexander *et al.* (1977), although in a summary of collected data from the literature, Bunn *et al.* (1977), reported an incidence of cerebral metastases of only 4·9% in patients after nitrosourea treatment but 24·8% after other forms of chemotherapy.

Set against the gains for the chemotherapy series in the present study was the much more frequent toxicity. This affected 83% of the patients, compared with less than a third of the patients in the radiotherapy series, even when adverse reactions to all the palliative measures received by both series are included. The most frequent toxic effects in the chemotherapy series were nausea and vomiting, which were reported in 71% of the patients. Depression of marrow function occurred in 54%, and not infrequently created problems of management. Adverse reactions frequently led to the abandonment of chemotherapy or modification of the dosage schedule. Even so, 38 of all 101 patients who started the allocated course of chemotherapy completed the prescribed 10 pulses (some with minor modifications) and, in the event, 19 received extra pulses because they were responding, and even at 12 months 12 were still receiving pulses. It is usual for some degree of toxicity to be associated with such chemotherapy regimens and the present treatment was designed to be acceptable for outpatient use. In general this proved to be the case.

In diseases where long-term survival is infrequent, the quality of life is particularly important but difficult to measure satisfactorily. Although the patients in the chemotherapy series frequently reported symptoms for hours after a pulse of chemotherapy, the assessments of the general condition, the degree of physical activity and respiratory function by a clinical grading showed no important difference between the 2 series at any time during the year. Of the survivors at one year, a substantial proportion were in good general condition, physically active and without undue dyspnoea on exertion.

We are unable to make a detailed comparison of survival in our patients and those from other series, as methods of staging were dissimilar. However, the median survival observed here is not as good as for a few other series reported on small numbers of patients. These are around or slightly in excess of 12 months (Broder *et al.*, 1977). Cases in this present series were designated "limited disease" without taking into account the results of isotope screening of liver and brain, or of marrow examinations, even where available. (Not all participating centres were able to carry out these examinations on all patients.) Some would undoubtedly have been staged as "extensive" if these procedures had been undertaken routinely. Chemotherapy was begun 3 weeks after the radiation treatment, thus entailing a delay of around 6 weeks from the start of the radiation to the start of cytotoxic drugs. This delay may have had an adverse effect on survival. We are currently investigating this possibility in the third MRC small-cell study.

Finally, a word of caution about the 3-drug schedule used in this present investigation is necessary. Before the series reported here, it had previously been tested in a pilot study in 50 patients with more extensive disease, and found to have an acceptable level of toxicity. However, in the new MRC study (Third MRC small-cell study) this same radiotherapy plus chemotherapy regimen has probably led to some deaths associated with intractable vomiting and dehydration. There have also been deaths attributed to toxicity after the first pulse of chemotherapy in patients who were allocated the same drugs in a regimen which differs only in that the radiotherapy is preceded by 2 pulses of chemotherapy at intervals of 3 weeks. However, increasing the fluid intake and giving the patient sodium bicarbonate tablets for 48 h starting on the day of each chemotherapy treatment appear to have reduced or eliminated the risk.

In conclusion, this study has shown that a 3-drug regimen of cyclophosphamide, methotrexate and CCNU, given after a relatively low dose of radiotherapy to the primary lesion, has improved the survival during 12 months' follow-up.

The following physicians, radiotherapists and pathologists co-operated in the study:

*Bristol*: Dr H Eckert, Mr N. C. D. Pizey; *Cambridge*: Dr V. Baker, Prof. N. M. Bleehen, Dr P. G. I. Stovin, Dr C. R. Wiltshire; *Cardiff*: Dr G. Anderson, Dr S. G. Cotton, Dr B. Davies, Dr T. J. Deeley, Dr G. S. Kilpatrick, Dr R. Seal, Dr A. Seaton, Dr P. Smith; *Durham*: Dr J .E. Ennis, Dr G. S. Graham, Dr A. L. Hovenden, Dr J. S. Law; *Glasgow*: Dr J. C. J. L. Bath, Dr R. A. Burnett, Dr J. Cuthbert, Dr R. J. Cuthbert, Dr B. R. Hillis, Dr G. Johnston, Dr J. W. Kerr, Dr A. W. Lees, Dr I. McHattie, Dr A. R. Russell, Dr B. H. R. Stack, Dr K. R. Urquhart, Dr E. R. Watson, Dr H. Yosef; *Hammersmith*: Dr C. G. McKenzie, Dr G. W. Poole, Dr P. Stradling; *King's College*: Dr D. M. Brinkley, Dr B. A. Hollis, Dr P. Hugh-Jones, Mr A. M. Macarthur; *Middlesex, Ashford and Mount Vernon*: Dr M. H. Bennett, Prof. R. J. Berry, Dr W. C. D. Richards; *Newcastle*: Dr A. A. Brace, Dr R. A. L. Brewis, Dr W. K. Cowan, Dr R. G. B. Evans, Dr C. D. Jobling, Dr O. M. Koreich, Dr J. R. Lauckner, Dr P. O. Leggat, Dr I. MacLeod, Dr R. T. H. Shepherd, Dr B. J. Smith, Dr A. R. Somner, Dr E. A. Spriggs, Dr A. J. Watson; *Norwich*: Dr H. de C. Baker, Dr A. H. C. Couch, Dr B. D. W. Harrison, Dr A. W. Jackson, Mr W. F. Kerr, Dr M. J. Ostrowski, Dr J. H. Rack, Dr P. F. Roberts, Mr B. A. Ross; *Oxford*: Dr R. J. Adam, Dr J. M. Black, Dr W. S. Hamilton, Dr E. A. Hills, Dr E. O. S. Hope, Dr F. A. L. Kircher, Dr A. H. Laing, Dr D. J. Lane, Dr C. R. Newman, Dr A. O. Robson; *Plymouth*: Dr J. M. Brindle, Dr R. A. B. Drury, Dr A. C. Hunt, Dr W. Scarratt, Dr J. E. Scoble, Dr G. Sheers; *SE RHA*: Dr R. H. Andrews, Dr S. R. Drake, Dr M. Farquharson, Dr G. B. Forbes, Dr A. G. Gibson, Mr A. Golebiowski, Dr D. G. Jenkins, Dr J. Spencer Jones, Dr P. Matheson, Dr J. Pollert, Dr H. Wilson; *Sheffield*: Dr P. Huck, Dr M. Ross; *Southampton*: Dr P. E. Bodkin, Dr R. B. Buchanan, Dr R. C. Godfrey, Dr H. MacDonald, Dr G. M. Sterling, Dr A. E. Tattersfield, Prof. D. H. Wright; *Sunderland*: Dr E. L. Feinmann, Dr K. A. Irvine, Dr S. Nariman, Dr J. H. Rolland Ramsay, Dr A. B. White; *Teesside*: Dr P. Ryan, Dr T. Skeoch, Dr H. I. Williams; *Yorkshire*: Mr L. Campbell-Robson, Dr N. Chakrabarti, Mr J. S. Davidson, Dr W. Davidson, Dr W. H. Helm, Prof. C. A. Joslin, Dr H. S. Kellett, Dr A. J. King, Mr E. R. Lecutier, Dr D. Mackinnon, Dr D. K. Stevenson, Dr J. Stone, Dr G. W. Storey, Prof. R. L. Turner, Dr A. J. Ward.

Dr K. F. W. Hinson was the reference pathologist for the study.

The trial was co-ordinated in the Medical Research Council Tuberculosis and Chest Diseases Unit by Dr L. E. Hill assisted by Mr R. J. Stephens.

### APPENDIX

TABLE.—*Biological equivalents*

| Overall time in days | Fractionation pattern (f = fraction) | | |
|---|---|---|---|
| | 5f/week | 3f/week | 2f/week |
| 11 | 10 f: 2650 rad | 6 f: 2340 rad | 4 f: 2160 rad |
| 18 | 15 f: 3000 rad | 9 f: 2655 rad | 6 f: 2430 rad |
| 25 | 20 f: 3460 rad | 12 f: 3060 rad | 8 f: 2800 rad |
| 32 | 25 f: 3750 rad | 15 f: 3330 rad | 10 f: 3050 rad |

Notes:
1. These doses were intended to be "tumour doses", *i.e.* mid-line doses when parallel opposed fields are used.
2. These doses were considered to represent 80–85% of normal tissue tolerance.
3. An allowance of 13% was made between 5 treatments per week and 3 treatments per week.
4. An allowance of 23% was made between 5 treatments per week and 2 treatments per week.
5. These doses were suitable for the volumes in the chest that are usually treated when parallel opposed fields of 150 $cm^2$ to 300 $cm^2$ are applied.

### REFERENCES

ALEXANDER, M., GLATSTEIN, E. J., GORDON, D. S. & DANIELS, J. R. (1977) Combined modality treatment of oat cell carcinoma of the lung: a randomised trial. *Cancer Treat. Rep.*, **61**, 1.

BERGSAGEL, D. E., JENKIN, R. D. T., PRINGLE, J. F. & 4 others (1972) Lung cancer: clinical trial of radiotherapy alone *vs* radiotherapy plus cyclophosphamide. *Cancer*, **30**, 621.

BRODER, L. E., COHEN, M. H. & SELAWRY, O. S. (1977) Treatment of bronchogenic carcinoma. II small cell. *Cancer Treat. Rev.*, **4**, 219.

BUNN, P. A., COHEN, M. H., IHDE, D. C., FOSSIECK, B. E., MATTHEWS, M. J. & MINNA, J. D. (1977) Advances in small cell carcinoma. *Cancer Treat. Rep.*, **61**, 333.

DEELEY, T. J. (1966) A clinical trial to compare two different tumour dose levels in the treatment of advanced carcinoma of the bronchus. *Clin. Radiol.*, **17**, 299.

Durrant, K. R., Berry, R. J., Ellis, F., Ridehalgh, F. R., Black, J. M. & Hamilton, W. S. (1971) Comparison of treatment policies in inoperable bronchial carcinoma. *Lancet*, i, 715.

Eagan, R. T., Maurer, L. H., Forcier, R. J. & Tulloh, M. (1973) Combination chemotherapy and radiation therapy in small cell carcinoma of the lung. *Cancer*, **32**, 371.

Fox, W. & Scadding, J. G. (1973) Medical Research Council comparative trial of surgery and radiotherapy for the primary treatment of small celled or oat celled carcinoma of the bronchus. 10-year follow-up. *Lancet*, ii, 63.

Hansen, H. H., Muggia, F. M., Andrews, R. & Selawry, O. S. (1972) Intensive combined chemotherapy and radiotherapy in patients with non-resectable bronchogenic carcinoma. *Cancer*, **30**, 315.

Hansen, H. H., Selawry, O. S. Simon, R. & 4 others (1976) Combination chemotherapy of advanced lung cancer. A randomised trial. *Cancer*, **38**, 2201.

Høst, H. (1973) Cyclophosphamide as adjuvant to radiotherapy in the treatment of unresectable bronchogenic carcinoma. *Cancer Chemother. Rep.* (Suppl.), **4**, 161.

Kreyberg, L., Leibow, A. A. & Uehlinger, E. A. (1967) *Histological typing of lung tumours. W.H.O.*,

Livingston, R. B. (1976) Nitroso urea combinations in lung cancer. *Cancer Treat. Rep.*, **60**, 757.

Medical Research Council (1966) Comparative trial of surgery and radiotherapy for the primary treatment of small celled or oat celled carcinoma of the bronchus. *Lancet*, ii, 979.